# Chronic Spinal Cord Injury Regeneration with Combined Therapy Comprising Neural Stem/Progenitor Cell Transplantation, Rehabilitation, and Semaphorin 3A Inhibitor

**DOI:** 10.1523/ENEURO.0378-23.2024

**Published:** 2024-02-09

**Authors:** Takashi Yoshida, Syoichi Tashiro, Narihito Nagoshi, Munehisa Shinozaki, Takahiro Shibata, Mitsuhiro Inoue, Shoji Ogawa, Shinsuke Shibata, Tetsuya Tsuji, Hideyuki Okano, Masaya Nakamura

**Affiliations:** ^1^Departments of Rehabilitation Medicine, Keio University School of Medicine, Shinjuku-ku, Tokyo 160-8582, Japan; ^2^Orthopaedic Surgery, Keio University School of Medicine, Shinjuku-ku, Tokyo 160-8582, Japan; ^3^Physiology, Keio University School of Medicine, Shinjuku-ku, Tokyo 160-8582, Japan; ^4^Regenerative and Cellular Medicine Kobe Center, Sumitomo Pharma, Kobe, Hyogo 650-0047, Japan; ^5^Formulation Research & Development Laboratories, Sumitomo Pharma, Suita, Osaka 564-0053, Japan; ^6^Division of Microscopic Anatomy, Graduate School of Medical and Dental Sciences, Niigata University, Niigata-shi, Niigata 951-8510, Japan

**Keywords:** axon regeneration, cell transplantation, chronic phase spinal cord injury, rodent, semaphorin 3A inhibitor, treadmill training

## Abstract

Spinal cord injury (SCI) often results in various long-term sequelae, and chronically injured spinal cords exhibit a refractory feature, showing a limited response to cell transplantation therapies. To our knowledge, no preclinical studies have reported a treatment approach with results surpassing those of treatment comprising rehabilitation alone. In this study of rats with SCI, we propose a novel combined therapy involving a semaphorin 3A inhibitor (Sema3Ai), which enhances axonal regeneration, as the third treatment element in combination with neural stem/progenitor cell transplantation and rehabilitation. This comprehensive therapeutic strategy achieved significant improvements in host-derived neuronal and oligodendrocyte differentiation at the SCI epicenter and promoted axonal regeneration even in the chronically injured spinal cord. The elongated axons established functional electrical connections, contributing to significant enhancements in locomotor mobility when compared with animals treated with transplantation and rehabilitation. As a result, our combined transplantation, Sema3Ai, and rehabilitation treatment have the potential to serve as a critical step forward for chronic SCI patients, improving their ability to regain motor function.

## Significance Statement

Spinal cord injury (SCI) sometimes results in a fatal condition that often results in multiple disabilities, including paralysis. Numerous treatment approaches have been investigated for acute-phase SCI in rodents, but reports on the treatment of the chronic phase are limited. We compared three synergistic treatment elements—transplantation, rehabilitation, and medication—to combined transplantation and rehabilitation in rats with chronic SCI. Before treatment, both groups had insufficient hindlimb function in open-field walking. Hindlimb function with axonal regeneration of host tissue significantly improved in the combined transplantation, rehabilitation, and medication group, whereas the combined transplantation and rehabilitation group showed no such improvement. These results suggest that the triple combined treatment improves locomotor function and has the potential to improve gait appearance in chronic SCI patients.

## Introduction

Spinal cord injury (SCI) sometimes results in a fatal condition that can lead to paralysis, impacting a person's ability to live their life. Considerable research is underway to overcome this challenging aftereffect. Such research includes induced pluripotent stem cell-derived neural stem/progenitor cells (NS/PCs), which have demonstrated functional recovery in an animal model of acute-phase SCI (Nori et al., 2011; Salewski et al., 2015). The techniques for NS/PC transplantation are established and have recently gained more attention due to clinical trial research (Sugai et al., 2021). Although NS/PC transplantation alone has demonstrated functional recovery in acute phases, cell transplantation in the chronic phase, defined as 42 d after SCI (Kwon et al., 2013), has been reported to not result in any significant functional improvement (Nishimura et al., 2013).

To solve the issue of the chronic phase of SCI, combination therapies have been investigated and have shown moderate effects. In particular, the addition of rehabilitation training to transplantation demonstrated synergistic effects (Tashiro et al., 2016, 2018; Shibata et al., 2023). However, these previous studies used murine models, which do not typically exhibit cavity formation in response to SCI. Moreover, the combined grafted and rehabilitation animals did not show any significantly improved functional recovery compared with the animals treated with rehabilitation alone. Because rehabilitation therapy can be basically implemented in human patients, this result does not yet justify the clinical application of NS/PC transplantation as an additional therapeutic option for the chronic phase of SCI. Thus, further combinatory treatments are required, such as pharmacological agents (Tashiro et al., 2017).

Various axonal extension-inhibiting molecules have been identified, such as Nogo (GrandPré et al., 2000), chondroitin sulfate proteoglycan (CSPG) (McKeon et al., 1991), and semaphorin 3A (Sema3A) (Hashimoto et al., 2004). In particular, Sema3A has been reported to inhibit the extension of axonal regeneration after SCI, and an inhibitor of this molecule, xanthofulvin, can antagonize Sema3A function and enhance neurite extension (Kikuchi et al., 2003; Kumagai et al., 2003). Indeed, our previous study revealed that the application of Sema3A inhibitor (Sema3Ai) to SCI animal models resulted in significantly enhanced axonal regeneration and motor function recovery (Kaneko et al., 2006). Furthermore, Sema3Ai has demonstrated synergy with rehabilitation, resulting in additional locomotor function recovery compared with Sema3Ai treatment alone (Zhang et al., 2014). Although the Sema3Ais were applied in the acute-to-subacute phases of injury (Kaneko et al., 2006; Zhang et al., 2014), the strong effect on axonal elongation suggests the potential application of this tool for chronic injuries. Regarding the system of drug delivery, Sema3Ai are useful and less invasive because they are made using silicon sheets (Zhang et al., 2014), which contrasts with more difficult procedures in which a pump and duct are left under the skin for the continuous delivery of medicines such as chondroitinase ABC (Shinozaki et al., 2016).

Therefore, the aim of this study was to achieve more robust locomotor function through extensive axon regeneration via the addition of Sema3Ai to combined NS/PC transplantation and rehabilitation therapy in an animal model of chronic SCI.

## Materials and Methods

### Animals

Female nude rats (F344/NJcl-rnu/rnu, weight = 125–155 g, 8 weeks old; CLEA Japan) were used in this study. Rats were housed in a plastic cage (three or four per cage, width × depth × height = 24 cm × 42 cm × 24 cm) with hardwood sawdust bedding. Lighting conditions comprised a 12 h light/dark cycle (light on, 7:00–19:00; light off, 19:00–7:00). Food and water were freely available via the cage lid and a water bottle. All animal procedures were performed in accordance with the (author university) animal care committee's regulations.

### SCI model and grouping

All SCI animals were administered anesthesia via subcutaneous injection of 0.4 mg/kg medetomidine hydrochloride, 2 mg/kg midazolam, and 2.5 mg/kg butorphanol. After a T10 laminectomy, 220 kilodyne contusion injuries were applied toward the exposed dura mater by using a commercially available specialized spinal cord impactor (IH-0400, Precision Systems and Instrumentation), as in a previous study (Shinozaki et al., 2016). Next, 0.3-mm-thick artificial dura mater was placed just above the exposed dura mater, and the area was closed and fixed using a layer of muscles and 5-0 nylon sutures. Antibiotics (orbifloxacin, Sumitomo Pharma Animal Health) were injected for 1 week after the SCI. All injured rats received daily manual bladder evacuations until their urinary function recovered. The Basso–Beattie–Bresnahan (BBB) locomotor rating scale (Basso et al., 1995) and body weight were measured once a week, and their changes over time were plotted. At 42 d past injury (DPI), animals with a BBB score of 8 or below (not able to support individual weight) and 2 or above (to avoid injury from rehabilitation) were selected (30 out of the 63 SCI model rats). All of these rats selected at 42 DPI were balanced-randomized so that the average BBB scores and body weights were approximately equal at each time point until 46 DPI when dividing them into two groups at the time of SCI (*n* = 15 per group, Fig. 6*A*,*B*).

### NS/PC culture

The human umbilical cord blood–derived induced pluripotent stem cell (iPSC) line YZWJs513 was used for transplantation in this study (Umekage et al., 2019). NS/PCs were prepared from a Good Manufacturing Practice (GMP)-grade cell processing facility at the Center for iPS Cell Research and Application in Kyoto University (CiRA) and stock frozen. Six days before the transplantation procedure, the NS/PCs were quickly defrosted and cultured to neurospheres in suspension culture in a serum-free medium until the transplantation procedure. The neurospheres were treated with 10 μm *N*-[*N*-(3,5-difluorophenacetyl)-l-alanyl]-*S*-phenylglycine t-butyl ester (DAPT; D5942, Sigma-Aldrich), which is a gamma-secretase inhibitor, 1 d before the transplantation (Okubo et al., 2018).

### Treatment procedure

NS/PCs for transplantation were injected 1 mm rostrally and caudally from the center of the SCI on 49 DPI. Single injections of NS/PCs were diluted to approximately 5 × 10^5^ cells in 2 μl at each location. The injection was performed using a 5 µl syringe (87930, Hamilton Company) and stereotaxic microinjector (KDScientific Legato310, Muromachi Kikai) at a rate of 1 μl/min, and the needle was left in place for 3 min, as in a previous study (Hashimoto et al., 2023).

**Figure 1.**
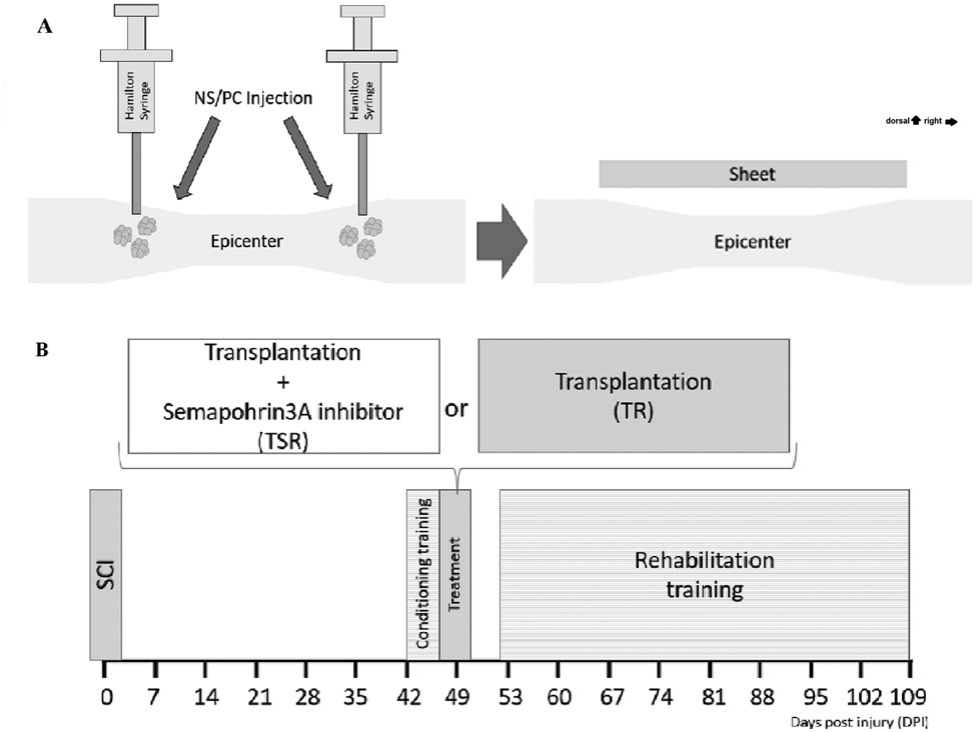
Schematic image of the treatment procedure and the experimental flow. ***A***, Schematic image of the treatment procedure; NS/PCs for transplantation were injected rostrally and caudally from the epicenter, and then a sheet of Sema3Ai or a placebo sheet was placed over the SCI epicenter. ***B***, Schematic image of the experimental flow; SCI models were divided into two groups, ensuring a close match in terms of mean BBB score and body weight up to 46 DPI. A 5 d regimen of conditional training was started on 42 DPI and ended on 46 DPI. The treatment procedure was administered on 49 DPI, followed by rehabilitation training, which began on 53 DPI and continued for 8 weeks.

Vinaxanthone, which has demonstrated physicochemical properties equivalent to xanthofulvin (Zhang et al., 2014), was used as Sema3Ai in this study. Vinaxanthone was administered using 0.3-mm-thick continuous-releasing matrix silicone sheets (SM-345431), a drug delivery system that was developed in a previous report (Zhang et al., 2014) and shows consistent release of the drug. The silicone sheets were trimmed into 2.5-mm-square pieces and placed over the SCI epicenter instead of artificial dura mater when the transplantation procedure was finished (Fig. 1*A*). The difference between the transplantation, Sema3Ai, and rehabilitation (TSR) and transplantation and rehabilitation (TR) groups was whether these silicone sheets contained Sema3Ai or not.

All of the treatments were performed under anesthesia induced via subcutaneous injection of 0.4 mg/kg medetomidine hydrochloride, 2 mg/kg midazolam, and 2.5 mg/kg butorphanol. Antibiotics (orbifloxacin) were injected for 3 d after the treatment procedure.

### Rehabilitation

The rehabilitation process was divided into two interventions: rehabilitation training for treatment and conditioning training to restore the fitness that may have been lost due to disuse and to help the rats acclimate and transition smoothly into rehabilitation training. A treadmill training machine (LE8706RTS, Panlab) was used for both training types. The training space was constructed with a 400 mm × 90 mm treadmill belt zone. This machine is equipped with an electrical grid zone at the end of each lane, but we occupied this area with paper obstacles to facilitate running so as not to use the electrical stimulus at this zone (Shibata et al., 2021). All rats underwent rehabilitation by voluntary quadrupedal locomotion on a flat treadmill for 20 min per day.

Five days of conditional training were started on 42 DPI and ended on 46 DPI before initiation of the treatment at a speed of 1 cm/s every day. Rehabilitation training started on 53 DPI, which was 4 d after the treatment procedure. Treatment rehabilitation continued for 8 weeks, as in a previous study (Tashiro et al., 2016). The speed was set to 1 cm/s for the first week and it was then increased by 1 cm/s every 2 weeks until the training ended (i.e., 1 cm/s in the first and second weeks, 2 cm/s in the third and fourth weeks).

### Behavioral testing

Hindlimb locomotor function was evaluated every week and immediately before the conditional training, rehabilitation training, and treatment procedure with the BBB locomotor rating scale (Basso et al., 1995), which is assessed in an open-field using a 21-point scale. The investigators conducting the scoring were blinded to the grouping information. Video for quantitative walking analysis was recorded on 109 DPI on a treadmill (DigiGait, Mouse Specifics) at a speed of 5 cm/s, and locomotion abilities were assessed by measuring the stride length, paw angle, stride duration times, and swing duration ratio of hindlimbs with ImageJ (ImageJ 1.53e, National Institutes of Health), which have been measured in the previous studies to indicate treatment efficacy (Hashimoto et al., 2023; Shibata et al., 2023).

### Motor evoked potential

Motor evoked potentials (MEPs) were recorded using a Neuropack S1 MEB-9402 (Nihon Kohden) on 109 DPI. MEPs were monitored under anesthesia induced using a subcutaneous injection of 0.2 mg/kg medetomidine hydrochloride, 2 mg/kg midazolam, and 2.5 mg/kg butorphanol tartrate. Th2 laminectomy was performed, and electrical stimulation was applied from the level of the spinal cord with a wired electrode. MEP signals were detected from the muscle belly and tendon of the quadriceps muscle by needle electrodes. The intensity of the stimulus was set to 5.0 mA. Stimulus duration and interval were set to 0.2 ms and 1 ms, respectively. The latency, amplitude, and duration of the induced potentials were recorded (Hashimoto et al., 2023).

### Weight measurement

Body weight was measured after BBB scoring every week and just before the conditional training, rehabilitation training, and treatment procedure. Gastrocnemius muscle was sampled after anesthetized rats were transcardially perfused with 0.9% sodium chloride with 5,000 units of heparin sodium and weighed.

#### Histological analysis and quantification

Spinal cord tissue from the SCI epicenter was collected for staining from all the models that completed rehabilitation training on 110 DPI (TSR *n* = 15, TR *n* = 15). Additional spinal cord tissue from the SCI epicenter was collected from one of the individuals who had an SCI but could not integrated into a therapeutic intervention on 49 DPI. The spinal cord was immersed overnight in 4% paraformaldehyde, overnight in 10% sucrose, and for 5 d in 30% sucrose. The target part of the spinal cord was embedded in FSC 22 Blue frozen section medium (Leica Biosystems) and frozen with liquid nitrogen.

Six of the 15 animals in each group were sectioned in the sagittal direction at a thickness of 16 µm, while the remaining nine were sectioned in the axial direction at a thickness of 20 µm using a cryostat (CM3050S, Leica Biosystems). The rostral and caudal edges of the SCI epicenter were defined based on the sections showing no normal gray matter from immunohistochemical (IHC) staining of axial sections for glutamic acid decarboxylase 67 (GAD67; 1:500, MAB5406, Merck), which stains normal gray matter. For quantification, individuals suitable for tissue evaluation were selected from each group and compared (TSR: *n* = 4, TR: *n* = 4).

Next, the following primary antibodies were used for IHC: human nuclear antigen (HNA; 1:100, MAB4383, Merck), HuC/HuD (ELAVL3/4; 1:100, A-21271, Thermo Fisher Scientific), adenomatous polyposis coli (APC; 1:300, OP80, Merck), Ki67 (1:1,000, ab16667, Abcam), glial fibrillary acidic protein (GFAP; 1:2,000, 16825-1-AP, Proteintech), STEM123 (1:1,000 Y40420, Takara Bio), STEM121 (1:100, Y40410, Takara Bio), Ser41 phosphorylated growth-associated protein 43 (pGAP43; 1:100, ab194929, Abcam), Ser96 pGAP43 (1:1,000, 18-10H-9H, Wako), neurofilament heavy polypeptide (NF-H; 1:300, ab8135, Abcam), 5-HT (20079, 1:2,000, ImmunoStar), NG2 (05-710, 1:1,000, Merck), and Sema3A (1:1,000, ab23393, Abcam).

Finally, Alexa Fluor–conjugated secondary antibodies were used with Hoechst 33258 (10 μg/ml, Merck). Images were acquired with a fluorescence microscope (BZ-X710; Keyence) and a confocal laser scanning microscope (LSM 780; Carl Zeiss). Basically, each of the comparable IHC images was stained and imaged in the same process. Axial areas for volume calculation were measured by ImageJ, independent of the person performing the measurement. Artifacts were removed using the rolling ball algorithm (Sternberg, 1983), and areas were calculated automatically. Three areas, namely, the epicenter and rostral and caudal edges, were measured and their volumes were calculated as truncated cones for comparison. The numbers of neurons and oligodendrocytes were counted with HNA and ELAVL3/4 or APC at three regions randomly captured within axial sections at 63-fold magnification as previously reported (Shibata et al., 2023). The NF-H length was measured in five areas of the SCI epicenter of each rat based on 63-fold images and averaged. Additionally, the values were averaged in each group and compared (Shibata et al., 2023).

### Experimental design and statistical analysis

All experiments were performed according to the process shown in Figure 1*B*.

All numbers used for quantitative analysis are noted in the figure legends.

Statistical analysis was performed with R: A Language and Environment for Statistical Computing (version 4.3.1, R Foundation for Statistical Computing) and EZR (Kanda, 2013). All data are presented as the mean ± standard error of the mean. Differences were considered significant at *p* < 0.05.

## Results

### Fibrous scar and Sema3A were present at the SCI epicenter during the therapeutic intervention

**Figure 2.**
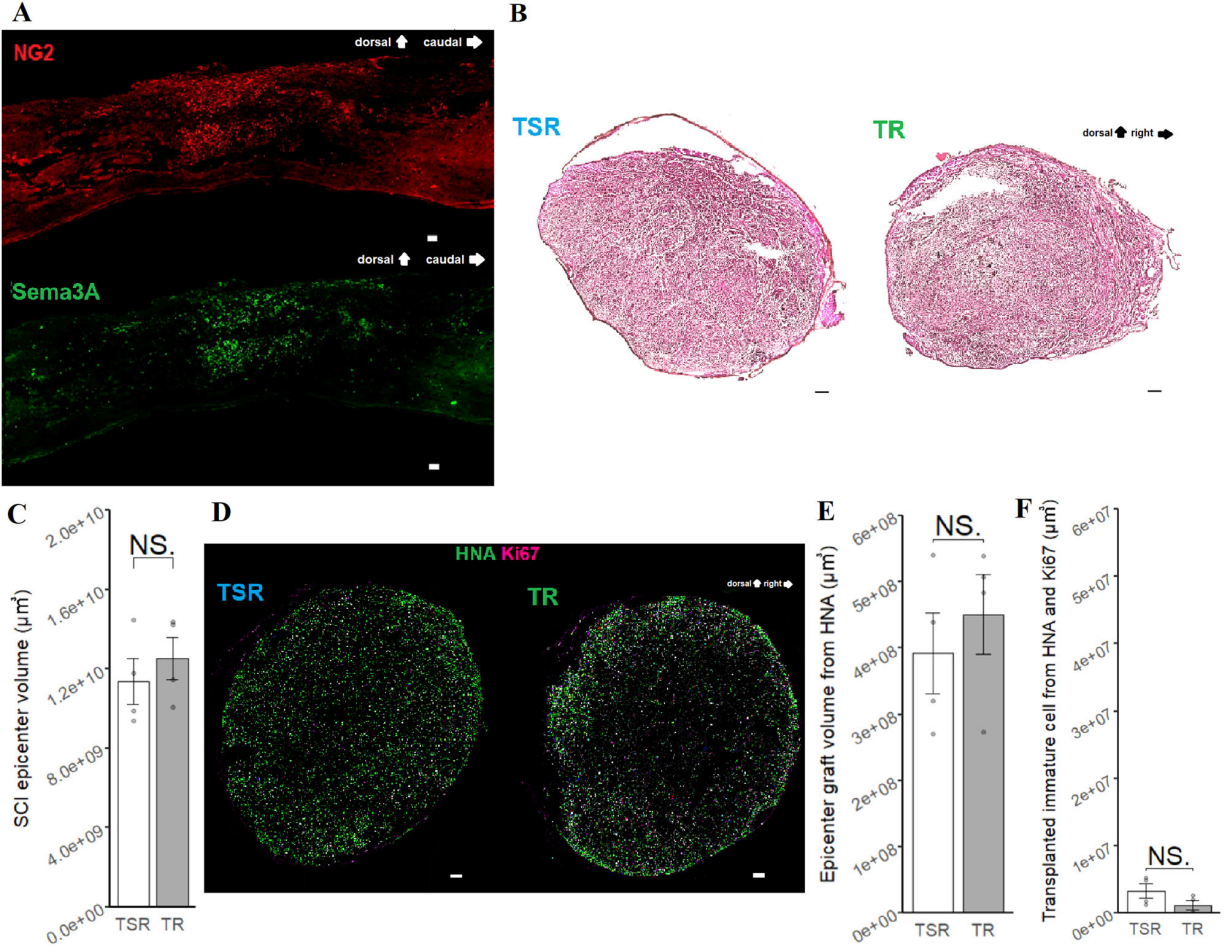
Comparison of fixation and immaturity of transplanted cells. ***A***, Sagittal-slice IHC of the SCI epicenter from a nontransplanted rat at 49 DPI, stained with NG2 and Sema3A. Scale bar, 100 μm. ***B***, Axial slice of the SCI epicenter with H&E staining of TSR group and TR group. Scale bar, 100 μm. Extended Figure 2-1 shows an axial slice of the SCI epicenter of a single rehabilitation group. ***C***, Comparison of the SCI epicenter volume with H&E staining at 110 DPI TSR (*n* = 4) and TR (*n* = 4), *p* = 0.72. ***D***, Axial-slice IHC of the SCI epicenter with HNA and Ki67 at 110 DPI. Scale bar, 100 μm. ***E***, Comparison of the transplanted volume with HNA. TSR (*n* = 4) and TR (*n* = 4), *p* = 0.73. ***F***, Comparison of the transplanted immature volume with Ki67. TSR (*n* = 4) and TR (*n* = 4), *p* = 0.14. All comparisons were tested with a two-sample *t* test. *p < 0.05, **p < 0.01, ***p < 0.005.

At the SCI epicenter on 49 DPI, the fibrous scar was confirmed with NG2 staining. The presence of Sema3A in the spinal cord was also confirmed on 49 DPI (Fig. 2*A*).

10.1523/ENEURO.0378-23.2024.f2-1 Figure 2-1 Axial-slice of the SCI epicenter with H&E staining of TR group and single rehabilitation group. Scale bar, 100 μm. Download Figure 2-1, TIF file.

### Sema3Ai did not affect the gross appearance of the SCI epicenter

To evaluate the effect of transplantation on the SCI epicenter after all rehabilitation (DPI 110), hematoxylin and eosin (H&E) staining was used to confirm the gross appearance (Fig. 2*B*). However, no significant difference was observed in the volume comparison of the SCI epicenter between the TSR and TR groups (Fig. 2*C*; *p* = 0.71). HNA staining was performed to determine the number of engrafted cells (Fig. 2*D*). The volume of transplanted cells calculated from the axial-slice area was not significantly different between the TSR and TR groups (Fig. 2*E*; *p* = 0.66).

### Sema3Ai did not affect the differentiation profiles of the transplanted cells

**Figure 3.**
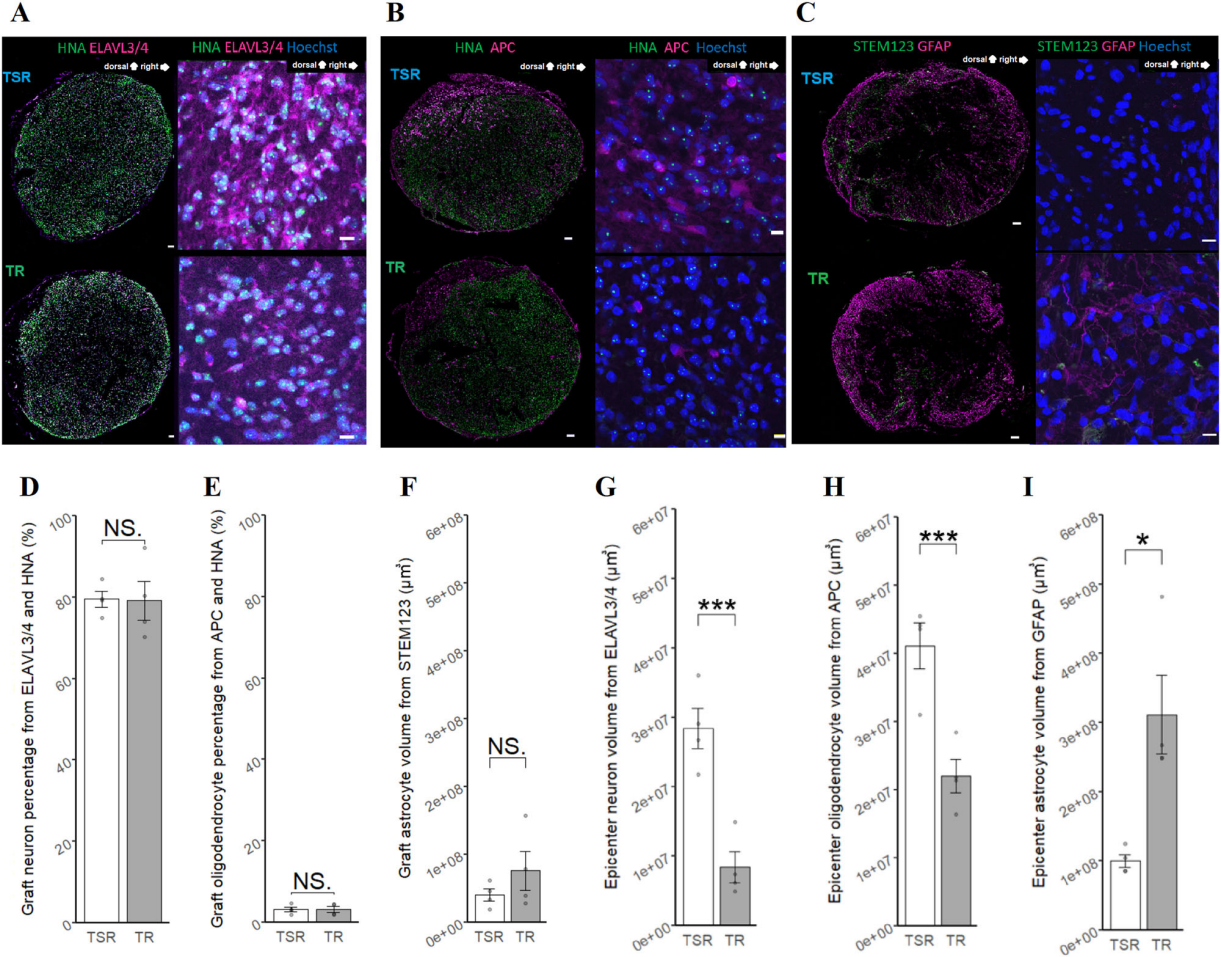
Differentiation of transplanted cells and formation of host tissue in the epicenter. ***A***, Axial-slice IHC of the SCI epicenter with HNA and ELAVL3/4. Scale bars: left side, 100 μm; right side, 10 µm. ***B***, Axial-slice IHC of the SCI epicenter with HNA and APC. Scale bars: left side, 100 μm; right side, 10 µm. ***C***, Axial-slice IHC of the SCI epicenter with GFAP and STEM123. Scale bars: left side, 100 μm; right side, 10 µm. ***D***, Comparison of the graft-derived differentiation percentage of neurons with HNA and ELAVL3/4. TSR (*n* = 4) and TR (*n* = 4), *p* = 0.94. ***E***, Comparison of the graft-derived differentiation percentage of oligodendrocytes with HNA and APC. TSR (*n* = 4) and TR (*n* = 4), *p* = 0.98. ***F***, Comparison of the graft-derived astrocyte volume with STEM123. TSR (*n* = 4) and TR (*n* = 4), *p* = 0.29. ***G***, Neuron volume comparison with ELAVL3/4. TSR (*n* = 4) and TR (*n* = 4), *p* = 0.0017, Cohen's *d* = 3.8. ***H***, Oligodendrocyte volume comparison with APC. TSR (*n* = 4) and TR (*n* = 4), *p* = 0.0038, Cohen's *d* = 3.2. ***I***, Astrocyte volume comparison with GFAP in the SCI epicenter. TSR (*n* = 4) and TR (*n* = 4), *p* = 0.010, Cohen's *d* = 2.6. All comparisons were tested with a two-sample *t* test. *p < 0.05, **p < 0.01, ***p < 0.005.

The proliferating cell marker Ki67 was used to distinguish undifferentiated cells in the NS/PC graft, but the stained volume was negligible (Fig. 2*D*,*F*; *p* = 0.099). Differentiation of NS/PCs was compared by calculating the percentage overlap between HNA and ELAVL3/4 for neurons (Fig. 3*A*) and between HNA and APC for oligodendrocytes (Fig. 3*B*). Additionally, the volume comparison of STEM123, which specifically labels human GFAP, was compared for astrocytes (Fig. 3*C*). Both neurons and oligodendrocytes showed similar mean values in the TSR and TR groups (Fig. 3*D*; *p* = 0.69, Fig. 3*E*; *p* = 0.60). Similarly, the volume comparison of STEM123 also showed similar mean values in the TSR and TR groups, akin to the findings observed for neurons and oligodendrocytes (Fig. 3*F*; *p* = 0.99).

### Sema3Ai did not positively modify the volume of host-derived astrocytes in the injured epicenter

Total neurons in the SCI epicenter were immunostained with anti-ELAVL3/4 (Fig. 3*A*), and oligodendrocytes were immunostained with anti-APC (Fig. 3*B*). The volumes of both cell types, which were calculated in axial slices, were larger in the TSR group than in the TR group (Fig. 3*G*; *p* = 0.017, Fig. 3*H*; *p* = 0.031). Astrocytes in the SCI epicenter were immunostained with anti-GFAP (Fig. 3*C*). In contrast to neurons and oligodendrocytes, astrocyte volume was smaller in the TSR group than in the TR group (Fig. 3*I*; *p* = 0.047).

### Sema3Ai induced axonal regeneration at the injured epicenter

**Figure 4.**
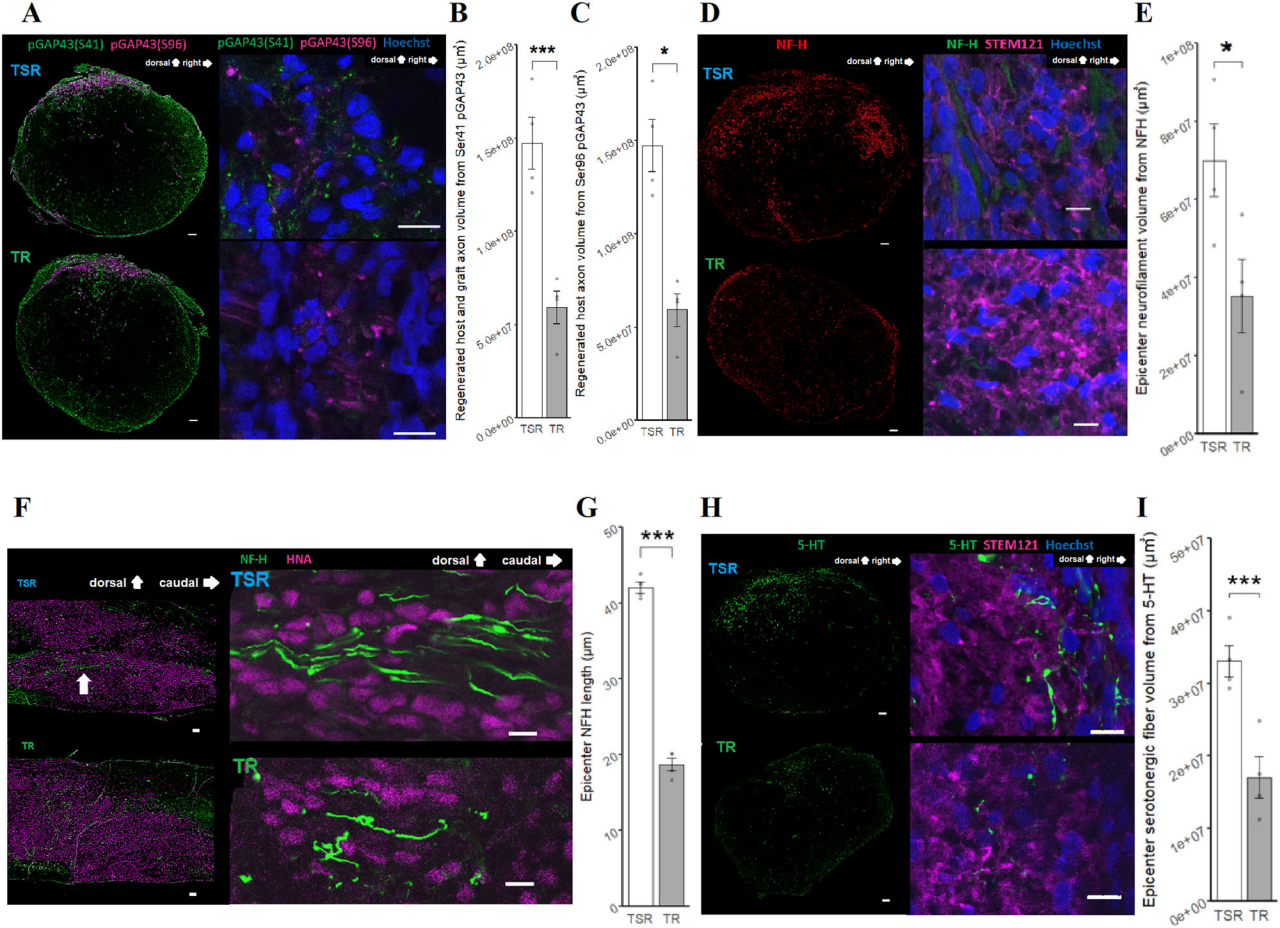
Comparative analysis of axon elongation. ***A***, Axial-slice IHC of the SCI epicenter with pGAP43(S41) and pGAP43(S96). Scale bars: left side, 100 μm; right side, 10 µm. ***B***, Total regenerated axon comparison with pGAP43(S41). TSR (*n* = 4) and TR (*n* = 4), *p* = 0.0018, Cohen's *d* = 3.8. ***C***, Host-derived regenerated axon comparison with pGAP43(S96). TSR (*n* = 4) and TR (*n* = 4), *p* = 0.034, Cohen's *d* = 1.9. ***D***, Axial-slice IHC of the SCI epicenter with neurofilament (NF-H) and STEM121. Scale bars: left side, 100 μm; right side, 10 µm. ***E***, Host-derived neurofilament volume comparison with NF-H. TSR (*n* = 4) and TR (*n* = 4), *p* = 0.039, Cohen's *d* = 1.9. ***F***, NF-H crossing the transplanted field (HNA) in a sagittal section. Scale bars: left side, 100 μm; right side, 5 µm. ***G***, Axon length comparison with NF-H. TSR (*n* = 4) and TR (*n* = 4), *p* = 0.00000081, Cohen's *d* = 15. ***H***, Axial-slice IHC of the SCI epicenter with 5-HT and STEM121. Scale bars: left side, 100 μm; right side, 10 µm. ***I***, Serotonergic fiber comparison with 5-HT. TSR (*n* = 4) and TR (*n* = 4), *p* = 0.0045, Cohen's *d* = 3.1. Extended Figure 4-1 shows graft axon expansion with STEM121 at the SCI epicenter. All comparisons were tested with a two-sample *t* test. *p < 0.05, **p < 0.01, ***p < 0.005.

Immunostaining for pGAP43 was used to evaluate axonal regeneration. To distinguish between host and graft factors, costaining was performed with the Ser41 pGAP43 antibody, which reacts against both human and rodent pGAP43, and Ser96 pGAP43, which reacts against rodent pGAP43 only (Fig. 4*A*). Both volumes of Ser41 pGAP43 and Ser96 pGAP43 were significantly larger in the TSR group than those in the TR group in the SCI epicenter (Fig. 4*B*; *p* = 0.0018, Fig. 4*C*; *p* = 0.034).

10.1523/ENEURO.0378-23.2024.f4-1 Figure 4-1 Graft axon expansion with STEM121 at the SCI epicenter. A, Graft axon expansion with STEM121 in TSR group at the SCI epicenter.in sagittal direction. Scale bar, 100 μm B, Graft axon expansion comparison with STEM121 volume TSR (n=4) and TR (n=4), p=0.62. The comparison was tested with a two-sample t-test. *p<0.05, **p<0.01, ***p<0.005. Download Figure 4-1, TIF file.

### Host-derived neurofilament increased at the injured epicenter

The NF-H expression volume was calculated from axial slices of the SCI epicenter to evaluate the neurofilament crossing between the rostral and caudal areas of the SCI epicenter (Fig. 4*D*,*E*). The TSR group showed a significantly larger volume of NF-H than the TR group (Fig. 4*E*; *p* = 0.049). Under high magnification, costained NF-H and STEM121 (human cytoplasm–specific antibody) did not overlap, as indicated by the NF-H antibody staining (Fig. 4*F*). Sagittal images showed that this host-derived NF-H crossed the transplanted field of HNA in the SCI epicenter (Fig. 4*F*). The average consecutive length of NF-H under high magnification was significantly longer in the TSR group than that in the TR group (Fig. 4*G*; *p* = 0.000075).

5-HT was costained with STEM121, a specific marker of human cytoplasm, to distinguish the origin (Fig. 4*H*). There was some colocalization, but most of the area did not overlap. The TSR group showed a significantly larger volume of 5-HT in the SCI epicenter than the TR group (Fig. 4*I*; *p* = 0.018).

### The TSR group showed a longer duration of MEP activation

**Figure 5.**
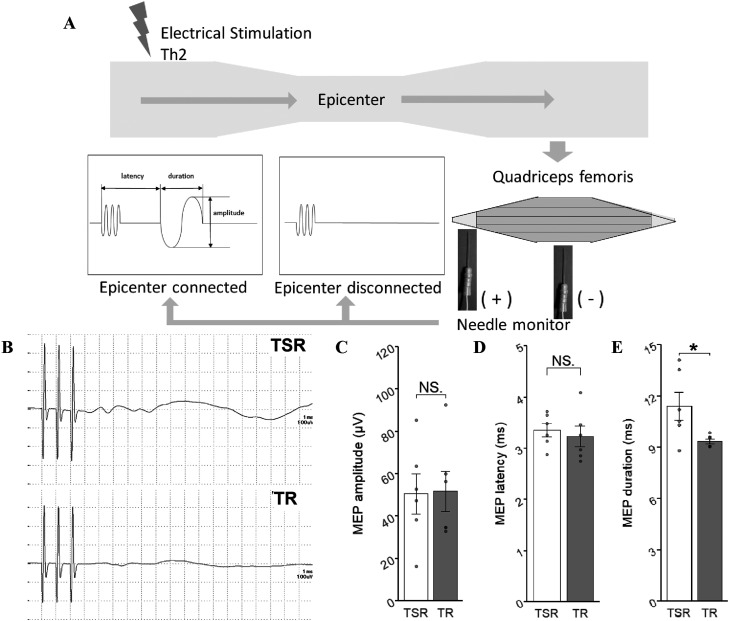
Image and comparative analysis of MEP. ***A***, Schematic image of MEP. ***B***, Representative MEP comparison. ***C***, MEP comparison with amplitude. TSR (*n* = 6) and TR (*n* = 6), *p* = 0.93. ***D***, MEP comparison with latency. TSR (*n* = 6) and TR (*n* = 6), *p* = 0.62. ***E***, MEP comparison with duration. TSR (*n* = 6) and TR (*n* = 6), *p* = 0.035. All comparisons were tested with a two-sample *t* test. *p < 0.05, **p < 0.01, ***p < 0.005.

MEPs were tested to confirm the electrical connection between the rostral and caudal edges of the SCI epicenter (Fig. 5*A*,*B*). The mean values for activated potential amplitude and latency were similar in the two groups (Fig. 5*C*; *p* = 0.93, Fig. 5*D*; *p* = 0.62). In contrast, activated potential duration was significantly longer in the TSR group (Fig. 5*E*; *p* = 0.035).

### The TSR group showed significantly greater locomotor recovery compared with the TR group

**Figure 6.**
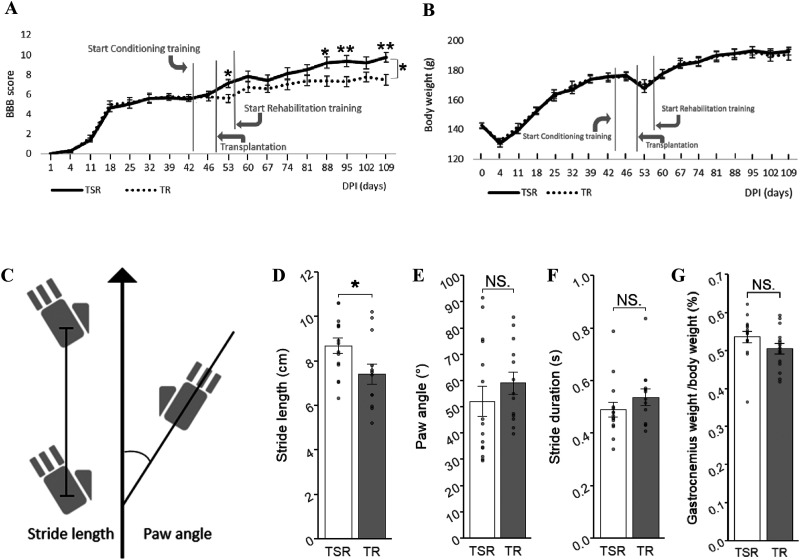
Transition and comparative analysis of locomotor abilities. ***A***, BBB score comparison. TSR (*n* = 15) and TR (*n* = 15). Repeated-measures ANOVA assuming sphericity. *p* = 0.018. Tukey’s multiple comparisons of means: 53 DPI, *p* = 0.013; 60 DPI, *p* = 0.14; 67 DPI, *p* = 0.15; 74 DPI, *p* = 0.20; 81 DPI, *p* = 0.14; 88 DPI, *p* = 0.016; 95 DPI, *p* = 0.0072; 102 DPI, *p* = 0.07; 109 DPI, *p* = 0.0031. Extended Figure 6-1 shows a comparison of BBB score with rehabilitation + Sema3Ai and rehabilitation. ***B***, Body weight transition. Body weight comparison TSR (*n* = 15) and TR (*n* = 15). Two-sample *t* test: 109 DPI, *p*-value = 0.583. ***C***, Schematic image of quantitative walking analysis. ***D***, Quantitative walking analysis with stride length. TSR (*n* = 15) and TR (*n* = 13), *p* = 0.026. ***E***, Quantitative walking analysis with paw angle. TSR (*n* = 15) and TR (*n* = 13), *p* = 0.34. ***F***, Quantitative walking analysis with stride duration time. *p* = 0.26. ***G***, Gastrocnemius weight/body weight. Two-sample *t* test *p*-value = 0.142. Comparisons in ***D–G*** were conducted with a two-sample *t* test. *p < 0.05, **p < 0.01, ***p < 0.005.

The BBB locomotor rating scale and quantitative gait analysis were used to examine the locomotor recovery and behavioral differences between the TSR and TR groups. The hindlimb movement of the TSR group was significantly improved compared with that of the TR group at the end of the whole rehabilitation process (Fig. 6*A*; *p* = 0.018, Table 1).[Table T2]

10.1523/ENEURO.0378-23.2024.f6-1 Figure 6-1 Comparison of BBB score with Rehabilitation + Sema3Ai (n=26) and Rehabilitation (n=26). Download Figure 6-1, TIF file.

**Table 1.** Correspondence table of BBB for each individual at 46 d when groups were divided and at 109 d when all interventions were completed

ID	46 DPI BBB	109 DPI BBB	ID	46 DPI BBB	109 DPI BBB
TSR1	5	10	TR1	5.5	7
TSR2	5.5	12.5	TR2	6	7
TSR3	5	9	TR3	5	9
TSR4	4	11	TR4	3.5	5
TSR5	4	6	TR5	3	3
TSR6	5	8	TR6	3.5	6
TSR7	6	10.5	TR7	6	8
TSR8	6	7	TR8	6	8
TSR9	5.5	8.5	TR9	6	5.5
TSR10	6	10	TR10	7	10
TSR11	7	12	TR11	7	7.5
TSR12	6	9	TR12	7	10
TSR13	8	10	TR13	6.5	9
TSR14	7	10	TR14	6.5	8.5
TSR15	9	12.5	TR15	7.5	8.5

**Table 2. T2:** Correspondence table of body weight for each individual at 46 d when groups were divided and at 109 d when all interventions were completed

ID	46 DPI weight	109 DPI weight	ID	46 DPI weight	109 DPI weight
TSR1	182	202	TR1	166	178
TSR2	174	196	TR2	176	200
TSR3	186	204	TR3	178	192
TSR4	166	178	TR4	172	180
TSR5	180	198	TR5	150	166
TSR6	184	204	TR6	172	178
TSR7	184	200	TR7	192	214
TSR8	194	190	TR8	196	202
TSR9	170	182	TR9	174	198
TSR10	186	210	TR10	180	198
TSR11	166	180	TR11	168	186
TSR12	172	184	TR12	186	198
TSR13	170	184	TR13	188	210
TSR14	166	182	TR14	168	184
TSR15	168	198	TR15	170	190

Quantitative gait analysis involved the measurement of stride length, paw angle, and stride duration time (Fig. 6*C–F*). Stride length was significantly longer in the TSR group than that in the TR group (Fig. 6*D*; *p* = 0.026). Paw angle and stride duration were not significantly different (Fig. 6*E*; *p* = 0.34, Fig. 6*F*; *p* = 0.26).

Body weights were measured weekly, and both groups showed largely the same trajectory (Fig. 6*B*, Table 2). To measure the use and muscle amount of the hindlimb, we compared the weight of the gastrocnemius muscle as a percentage of body weight after the rehabilitation procedure. However, there were no significant differences between the two groups (Fig. 6*G*; *p* = 0.142).

## Discussion

In this study, we applied combined Sema3Ai with NS/PC transplantation and quadrupedal treadmill gait rehabilitation therapy to rats in the chronic phase of SCI to explore its potential for additional recovery. The three-part treatment resulted in significant locomotor recovery compared with the dual treatment involving cell transplantation and treadmill gait training. We believe that our novel combined approach involving Sema3Ai opens new possibilities for the application of NS/PC transplantation to the treatment of chronic SCI patients because previous strategies involving NS/PCs and rehabilitation failed to significantly surpass the benefits of rehabilitation alone (Tashiro et al., 2016).

### Combined Sema3Ai, NS/PC transplantation, and rehabilitation therapy improved locomotor ability in chronic phase SCI via electrically connected host axon regeneration

The fibrous scar induced by SCI in this study was confirmed through NG2 staining (Jin et al., 2022). Additionally, the presence of Sema3A was detected by IHC in close proximity to the NG2-stained area (Fig. 2*A*), consistent with previous results (Pasterkamp et al., 1998).

To assess axonal regeneration, we used the nonreactive properties of Ser96 pGAP43 for rodents (Okada et al., 2021), as well as Ser41 pGAP43, which is reactive in both humans and rodents (Coggins and Zwiers, 1991), to distinguish the origin of the regenerated axons. If Ser41 pGAP43 showed a significant difference while Ser96 pGAP43 did not, it would indicate that the transplanted human NS/PCs were the source of the regenerated axons. However, our results showed that both Ser41 pGAP43 and Ser96 pGAP43 were increased in the TSR group compared with the TR group, suggesting that the major elongated nerve fibers observed were likely of host origin.

Next, we used a rodent-specific antibody for NF-H, which is a major component of mature axons (Hashimoto et al., 2023). We histologically observed a significant increase in neuronal fibers in the TSR group compared with the TR group. This finding supports the ability of Sema3Ai to induce host axonal elongation at the SCI epicenter, even in the chronic phase, and is consistent with previous reports that performed this treatment in acute-to-subacute SCI (Kaneko et al., 2006; Zhang et al., 2014).

To investigate which type of neurons elongated, we focused on serotonergic 5-HT–positive axons because they crossed the SCI epicenter in the acute phase of treatment (Kaneko et al., 2006). As expected, we observed a quantitative increase in the volume of serotonergic 5-HT–positive axons in the TSR group compared with the TR group. Because anti–5-HT antibodies react against both human and rodent serotonergic neurons, we costained with STEM121, a specific marker of human cytoplasm, to distinguish the species origin of the serotonergic fibers. We found a very limited overlap of STEM121 with 5-HT–positive neurons, leading us to conclude that the serotonergic fibers are primarily of host tissue origin. Overall, the addition of Sema3Ai promoted the regeneration of host-derived axons, particularly those with serotonergic properties.

MEP testing was performed to confirm connectivity between the rostral and caudal regions of the SCI epicenter. Regarding the shortest latency, no significant difference was observed between the TSR and TR groups (Fig. 5*B*), and the value was close to that of the few non-SCI models tested. This component probably corresponds to the original and intact connectivity that escaped damage from the SCI. Because it does not require repair, it is reasonable that this factor showed comparable results between the TSR and TR groups. In intact cases, MEPs have a large amplitude because all of the anterior cells of the spinal cord send signals simultaneously. However, in SCI models, most of the signal-sending cells are lost, resulting in a reduction in MEP amplitude (Nakatoh et al., 2001). The prolonged duration of the MEP can be interpreted as the waveform conveying a greater number of varieties of functional axons, including axons of different diameters, some that have regenerated and/or remyelinated to varying degrees, or those with synapses in the process of descending. The original large waveform can be considered to be dispersed in time due to these variations in regenerated axons (Fig. 5*B*). The fact that the amplitude was not significantly different between the two groups could support the idea that the signal dispersed in time, resulting in phase cancelation. This phenomenon indicates the presence of regenerated axons that are functional in both groups. The results suggest that the addition of Sema3Ai to the NS/PC transplantation and rehabilitation combination therapy contributes to the regeneration of functional axons in the chronic SCI setting. It is noteworthy that many of the present results are basically obtained from sections, but not from the animals. Strictly, such a procedure may not be suitable to represent the true variability and possibly also the values.

Regarding locomotor function, the TSR group showed a significant improvement in BBB score, starting from the fifth week of the rehabilitation training. After completion of all rehabilitation training sessions, the TSR group maintained a significantly higher BBB score, which was further supported by a significant increase in stride length. Based on the histologic and electrophysiologic assessments, we conclude that the regeneration of electrically connecting axons contributed to the locomotor improvement due to the triple combined therapy.

### Sema3Ai modified the composition of the host but not transplanted cell-derived neural cells in the SCI epicenter

In this study, the transplanted NS/PCs were observed to differentiate predominantly into neurons (Fig. 3*D*), but Sema3Ai did not significantly promote neuronal differentiation. In addition, the differentiation properties of oligodendrocytes (Fig. 3*E*) and astrocytes (Fig. 3*F*) were also comparable between the groups with and without Sema3Ai in the spinal cord. One possibility is that the transplanted cells are less mature than the host cells and do not therefore express receptors for Sema3A. This would mean that they are not influenced by Sema3Ai, although this specific scenario has not previously been reported in the literature. However, similar to our results, there is a report indicating that semaphorins secreted by transplanted olfactory ensheathing cells did not affect the cell properties (Reginensi et al., 2015). Therefore, it is plausible that the response of transplanted cells to Sema3A may differ from that of host cells. Regarding the safety issue, few transplanted cells expressed the proliferating cell marker Ki67, suggesting that Sema3Ai did not induce NS/PCs to remain immature within the injured spinal cord (Fig. 2*F*). However, Sema3Ai treatment significantly altered the composition of host neural cells, with a notable increase in the area of neurons and oligodendrocytes and a decrease in that of astrocytes (Fig. 3*G*,*I*). The observation that the volume of the transplanted area did not differ between the presence and absence of the Sema3Ai, contrary to the previous reports (Kaneko et al., 2006), could be influenced by the chronicity of the injured spinal cord which limits the simple treatment effect of Sema3Ai and the substantial increase in volume caused by the transplantation itself as observed in our new data from single rehabilitation in the chronic phase (Extended Fig. 2-1).

There could be several reasons for the elevated numbers of neurons and oligodendrocytes in the SCI epicenter. It is widely accepted that Sema3Ai administration to acute SCI rats can stimulate axonal regeneration (Kaneko et al., 2006; Hira et al., 2018). The present study confirmed the extension of newly formed axons and fibers stained with NF-H, as reported in previous studies. This led us to conclude that the extended axons that we observed were comparable to the previously reported extension achieved with Sema3Ai. In contrast, our therapeutic target was not the acute but the chronic phase of SCI. Although no previous reports have explicitly mentioned the observed increase in oligodendrocytes following Sema3Ai treatment, it has been reported that the promotion of remyelination supported axonal elongation (Zawadzka et al., 2022). Therefore, the remyelination observed in the current study could be one of the mechanisms of action of Sema3Ai on axon extension.

In contrast to the behavior of neurons and oligodendrocytes, astrocytes occupied a smaller area in the SCI epicenter of the TSR group compared with the TR group. This is consistent with a previous study that demonstrated a suppression in the area of GFAP-positive astrocytes at the lesion site when a single treatment of Sema3Ai was applied to middle cerebral artery–occluded rats during the subacute phase (Hira et al., 2018). Because the residual astrocytes in the chronic phase of SCI inhibited axonal elongation (Silver and Miller, 2004; Karimi-Abdolrezaee and Billakanti, 2012; Nathan and Li, 2017), our findings of astrocyte reduction in host tissues may indicate a contribution to functional recovery through neuronal plasticity.

Interestingly, the component that showed improvement with Sema3Ai treatment was mainly of host origin. The transplanted cells may have served as a scaffold in the SCI epicenter because previous studies have shown that motor function-related 5-HT fibers extend through the areas where transplanted cells are present (Hashimoto et al., 2023). Moreover, the Sema3Ai used in this study has the potential to enhance axon elongation, allowing them to traverse the scaffold created by the transplanted cells, as shown in Figure 4*F*. The absence of a difference in motor function between rehabilitation with inhibitors and rehabilitation alone during the chronic phase, as suggested by our new data, could also provide support (Extended Fig. 6-1). Consequently, this result could indicate that the addition of Sema3Ai to combined NS/PC transplantation and rehabilitation therapy improves the electrical connection between the rostral and caudal regions of the SCI epicenter, leading to locomotor recovery.

### Limitation

In this study, we observed host-specific axon outgrowth, which was attributed to the presence of specific antibodies targeting these axons. Notably, previous research has not investigated the origin of axon elongation in scenarios where neural stem/progenitor cells (NS/PCs) were transplanted 42 d after injury. This time frame, recommended by Kwon et al. (2013), is considered sufficient to enter the chronic phase of rehabilitation. In their study on spinal cord injury (SCI) during the chronic phase of rehabilitation, Tashiro et al. (2016) did not evaluate the extent of axon growth from transplanted cells. Similarly, Shibata et al. (2023) also overlooked this aspect in their investigation. As for the papers with transplants, Karimi-Abdolrezaee et al. (2010), Kumamaru et al. (2013), and, Kawabata et al. (2016) also did not evaluate transplant-derived axons either. In the paper on SCI in the chronic phase without rehabilitation, Hashimoto et al. (2023) have shown the existence of graft-derived axons through immunohistochemistry using STEM121. In this regard, the present study has also confirmed STEM121 at the SCI epicenter in Figure 4, *D* and *H*, and Extended Figure 4-1. However, it's important to note that STEM121 is not specific for axons. Although the transplanted cells were NSCs in the subacute phase, Lu et al. (2012) evaluated graft-derived axons by labeling the transplanted cells with GFP. However, this was not done in this study because it would have interfered with other evaluations. Further investigation is needed to determine what is important for the axonal extension of graft-derived axons and what kind of changes would be induced when the present system, using a Semaphorin3A inhibitor, is applied to it in future studies.

## Conclusion

This study showed for the first time that the addition of a third element, Sema3Ai, surpasses the efficacy of the best treatment regimen for the chronic-stage treatment of SCI, beating the previously reported results of combined NS/PC transplantation and rehabilitation therapy (Tashiro et al., 2016; Shibata et al., 2023). This improvement was mainly observed in terms of motor function and was attributed to the elongation and electrical connectivity of axons in the transplanted area. We believe that our tripartite treatment has potential as a treatment for SCI patients in the chronic phase that helps them regain their locomotor function.

## Synthesis

Reviewing Editor: Selva Baltan, Oregon Health & Science University Department of Surgery Decisions are customarily a result of the Reviewing Editor and the peer reviewers coming together and discussing their recommendations until a consensus is reached. When revisions are invited, a fact-based synthesis statement explaining their decision and outlining what is needed to prepare a revision will be listed below. The following reviewer(s) agreed to reveal their identity: Nick Jeffery, Andrei Sdrulla.

Dear Dr. Nagoshi,

Both reviewers have agreed that this study has merit however, they emphasized that for the data to strongly support the hypothesis experimental numbers and statistical analysis need to be improved. In addition, a proper control group and further clarification of figures are required.

## Author Response

Response to Reviewers

We truly appreciate the close attention and thoughtful comments of the editor and the reviewers, and greatly appreciate their time and efforts with regard to our manuscript "Chronic Spinal Cord Injury Regeneration with Combined Therapy Comprising Neural Stem/Progenitor Cell Transplantation, Rehabilitation and Semaphorin 3A Inhibitor" (eneuro-11-ENEURO.0378-23.2024R1). Below, we have addressed each of the comments provided by the editor and reviewers, outlining our responses. We believe that the careful consideration of your comments has significantly enhanced the quality of our manuscript, and we are optimistic that the revisions align with the standards of eNeuro for potential publication.

Reviewer 1

General comments

The rationale for this study is very strong - many commentators have supported the future use of combinatorial therapy for patients with spinal cord injury and the ability to intervene in the chronic phase would enable many more patients to benefit. The rationale for investigating Sema 3A inhibitors is strong and based on data from the authors.

On the more negative side, the data presented here to support the hypothesis is weak - mainly because it is so sparse - so few data points have been examined. This means that the discriminatory power of many of the analyses is vey low and therefore the conclusions may be unreliable.

It would be very helpful to see more clear data on the behavioral outcome. The true complexity of the outcome response can be difficult to fully interpret based on simple mean values +/- sem.

(Response)

We truly appreciate your comment. The response will be addressed to the specific comments below.

Specific comments (by line number

------------------------------------------------------------------------------------------

17, 28: the emphasis on fatality is probably a little stronger than warranted in line 17 - 'sometimes fatal' might be more accurate?

(Response)

We appreciate the reviewer's feedback, and in response, we have adjusted the expression accordingly.

" Spinal cord injury (SCI) is a sometimes results in a fatal condition that often results in multiple disabilities, including paralysis." (line 17)

" Spinal cord injury (SCI) is an often sometimes results in a fatal condition that can lead to paralysis, impacting a person's ability to live their life." (line 28)

------------------------------------------------------------------------------------------

88: this is not 100% clear. Did you match rats and then give each one of the pair one of the treatments? If so, please state more clearly. It would be helpful to see the weight and BBB scores of these animals at matching.

(Response)

We thank for the reviewer's comment. As you pointed out, the groups were divided so that the average of weight and BBB scores, which had previously been measured once a week, would approximately equal at each time point until 46 DPI shown in Fig. 6A and 6B. In response, we have added the explanation in the Material and Methods section accordingly, and added Table 1 and Table 2 of weight and BBB scores of animals at matching.

" These rats were matched based on the mean BBB score and weight for close tracking of the groups balanced-randomized so that the average BBB scores and body weights were approximately equal at each time point until 46 DPI when dividing them into two groups at the time of SCI (n=15 per group, Fig.6A, B)." (line 88-90)

------------------------------------------------------------------------------------------

103-104: again not 100% clear - 5X105 cells at each location, right?

(Response)

We appreciate the reviewer's feedback, and in response, we have added the expression accordingly.

" NS/PCs for transplantation were injected 1 mm rostrally and caudally from the center of the SCI on 49 DPI. Single injections of NS/PCs were diluted to approximately 5 × 105 cells in 2 μl at each location." (line 104-106)

------------------------------------------------------------------------------------------

121: what does 'conditional' training mean?

(Response)

We appreciate the reviewer's comment. Conditional training was included in the experiment to habituate to rehabilitation training and to ameliorate disuse induced declines in fitness. In response, we have added the explanation accordingly.

" The rehabilitation process was divided into two interventions: rehabilitation training for treatment and conditioning training to restore the fitness that may have been lost dues to disuse and to help the rats acclimate and transition smoothly into rehabilitation training." (line 122-124)

------------------------------------------------------------------------------------------

140-141: why were these aspects of locomotion selected for this analysis?

(Response)

We thank for the reviewer's comment. These parameters of gait analysis were chosen for their simplicity in quantification and because they had been used in previous papers (Hashimoto et al., 2023; Shibata et al., 2023). In response, we have added the explanation accordingly.

" Video for quantitative walking analysis was recorded on 109 DPI on a treadmill (DigiGait, Mouse Specifics, Inc.) at a speed of 5 cm/s and locomotion abilities were assessed by measuring the stride length, paw angle, stride duration times, and swing duration ratio of hindlimbs with ImageJ (ImageJ 1.53e, National Institutes of Health), which have been measured in the previous studies to indicate treatment efficacy (Hashimoto et al., 2023; Shibata et al., 2023)." (line 145)

------------------------------------------------------------------------------------------

161: how many spinal cords were processed in his way? If not all, how were those that were analyzed selected?

203-211: it seems from graphs in Figure 2 that these comparisons are based on a sample size of 3 in each group - is that correct? If so, these are VERY underpowered statistical comparisons and are unlikely to be illuminating because they are likely to be very unreliable. I don't really understand why there are so few datapopints - see comment about line 161 above.

210-218: same comment as above - see fig 3

222-227: as above

230-234: slightly better but still only 4 datapoints per graph / statistical comparison.

(Response)

We thank for the reviewer's comment. All the SCI epicenter was collected from the models which completed rehabilitation training (TSR: n=15, TR: n=15). Nine of the 15 animals in each group were sectioned in axial direction, while the remaining six were sectioned in the sagittal direction. However, recognizing the concern about the small number of comparisons, we calculated the sample size with a sufficient effect size as indicated by Cohen's d in each comparison with significant difference. Following the suggestion, we again compared the tissues quantification again with four tissue suitable for tissue evaluation, ensuring the maintenance of differences with a sufficient effect size. In addition, we have added the text and changed figure accordingly.

" Spinal cord tissue from the SCI epicenter was collected for staining from all the models that completed rehabilitation training on at 110 DPI for staining (TSR n = 15, TR n = 15)." (line 165-166)

" Six of the 15 animals in each group were sectioned in the sagittal direction at a thickness of 16 µm, while the remaining nine were sectioned in the axial direction at a thickness of 20 µm using with a cryostat (CM3050S, Leica Biosystems)." (line 172-174)

" For quantification, individuals suitable for tissue evaluation were selected from each group and compared. (TSR: n=4, TR: n=4)." (line 177-178)

" C, Comparison of the SCI epicenter volume with H&E staining at 110 DPI TSR (n=4) and TR (n=4), p=0.72." (line 556-557)

" E, Comparison of the transplanted volume with HNA. TSR (n=4) and TR (n=4), p=0.73. F, Comparison of the transplanted immature volume with Ki67. TSR (n=4) and TR (n=4), p=0.14. (line 558-559)

" D, Comparison of the graft-derived differentiation percentage of neurons with HNA and ELAVL3/4. TSR (n=4) and TR (n=4), p=0.94. E, Comparison of the graft-derived differentiation percentage of oligodendrocytes with HNA and APC. TSR (n=4) and TR (n=4), p=0.98. F, Comparison of the graft-derived astrocyte volume with STEM123. TSR (n=4) and TR (n=4), p=0.29. G, Neuron volume comparison with ELAVL3/4. TSR (n=4) and TR (n=4), p=0.0017, Cohen's d:3.8. H, Oligodendrocyte volume comparison with APC. TSR (n=4) and TR (n=4), p=0.0038, Cohen's d:3.2. I, Astrocyte volume comparison with GFAP in the SCI epicenter. TSR (n=4) and TR (n=4), p=0.010, Cohen's d:2.6." (line 566-573)

" B, Total regenerated axon comparison with pGAP43(S41). TSR (n=4) and TR (n=4), p=0.0018, Cohen's d:3.8. C, Host-derived regenerated axon comparison with pGAP43(S96). TSR (n=4) and TR (n=4), p=0.034, Cohen's d:1.9. D, Axial-slice IHC of the SCI epicenter with Neurofilament (NF-H) and STEM121. Scale bars: left side, 100 μm; right side, 10 µm. E, Host-derived neurofilament volume comparison with NF-H from STEM121. TSR (n=4) and TR (n=4), p=0.039, Cohen's d:1.9. F, Neurofilament (NF-H) crossing the transplanted field (HNA) in a sagittal section. Scale bars: left side, 100 μm; right side, 5 µm. G, Axon length comparison with NF-H. TSR (n=4) and TR (n=4), p=0.00000081, Cohen's d:15. H, Axial-slice IHC of the SCI epicenter with 5-HT and STEM121. Scale bars: left side, 100 μm; right side, 10 µm I, Serotonergic fiber comparison with 5-HT. TSR (n=4) and TR (n=4), p=0.0045, Cohen's d:3.1." (line 577-587)

------------------------------------------------------------------------------------------

198: not clear from which group this tissue came from. Is this tissue removed at surgery when transplanting? If not, there is no mention of animals euthanased at day 49 in the M&M, nor how they might have been selected.

(Response)

We thank for the reviewer's comment and apologize for insufficient explanation. Additional spinal cord tissue from the SCI epicenter was collected from one of the individuals which SCI had made but did not integrate into a therapeutic intervention on 49 DPI for Fig. 2A. In response, we have added the explanation accordingly.

Additional spinal cord tissue from the SCI epicenter was collected from one of the individuals which had a SCI but did not integrate into a therapeutic intervention on 49 DPI." (line 166-168)

------------------------------------------------------------------------------------------

180: how were the rostral and caudal edges defined? Not clear how the 'cone' was defined.

(Response)

We thank for the reviewer's comment. The rostral and caudal edge of SCI epicenter were defined based on the sections showing no normal gray matter from immunohistochemistry staining of axial slices for GAD67, which stains normal gray matter. In response, we have added the explanation accordingly.

" The rostral and caudal edges of the SCI epicenter were defined based on the sections showing no normal gray matter from immunohistochemical stry (IHC) staining of axial sections for glutamic acid decarboxylase 67 (GAD67) (1:500, MAB5406, Merck), which stains normal gray matter." (line 174-177)

------------------------------------------------------------------------------------------

181-186: were these counts / measures made by blinded investigators?

(Response)

We thank for the reviewer's comment. Quantification was performed by Image J automatically not to influence from the operator. In response, we have added the explanation accordingly.

" Axial areas for volume calculation ing volumes were measured by ImageJ, independent of the person performing the measurement. Artifacts were removed using the rolling ball algorithm (Sternberg, 1983), and areas were calculated automatically." (line 190-192)

" Sternberg, "Biomedical Image Processing," in Computer, vol. 16, no. 1, pp. 22-34, Jan. 1983, doi: 10.1109/MC.1983.1654163." (line 517-518)

------------------------------------------------------------------------------------------

203-204: what was measured? Is this just the diameter?

(Response)

We thank for the reviewer's comment. The volume of the SCI epicenter was measured for comparison as described in line 216-217.

------------------------------------------------------------------------------------------

254-256: this is inference (and therefore not necessarily true) and not results.

(Response)

We thank for the clarification. In response, we have removed the suggested sentence.

------------------------------------------------------------------------------------------

258-270 / Fig 6: the use of mean (+/- sem) can be quite difficult to interpret for BBB scores. It would be good to see a table of BBB scores at baseline (day 42) and day 109 for rats in both groups (ie scores with numbers of animals in each group scoring each value on the BBB score). This would be far mor informative for readers to be able to make comparisons between outcomes in the two groups.

(Response)

We thank for the reviewer's comment. In response, we have added a correspondence table of BBB (Table 1) and body weight (Table 2) for each individual at 46 days when groups were divided and at 109 days when all interventions were completed.

------------------------------------------------------------------------------------------

-

Reviewer 2

This rat study compares the effects of a semaphoring inhibitor (Sema3Ai, or xanthofulvin) in a spinal cord injury model where induced pluripotent stem cells were transplanted, in conjunction with rehabilitative therapy. Sema3Ai or vehicle were added 49 days after injury, the histology, electrophysiology, and behavior were used to determine effects. The overall conclusion of the study is that Sema3Ai improved regeneration and enhanced functional recovery compared with vehicle. The study is significant as we lack effective treatments for spinal cord injury, especially in the chronic phase.

The manuscript is well written, and the figures are clear and well documented in the legends and results sections. The authors chose rats with similar BBB scores, resulting in a more homogeneous SCI sample, as variable injuries would increase outcome variance.

------------------------------------------------------------------------------------------

1) The results are difficult to interpret since there is a lack of control with no stem cell transplantation, and the prior studies examined acute and subacute phases of injury. There needs to be a better justification for not including a group without transplantation, given the lack of prior studies investigating the effects of Sema3Ai on chronic injury.

(Response)

We thank for the reviewer's comment. In implementing the present study, we have decided not to include a group without transplantation, which stands for the rehabilitation and Sema3Ai combined group, for the following reasons. Our new data showed no difference in motor function between the combination of rehabilitation and Sema3Ai in the chronic phase of SCI compared to rehabilitation alone (Figure 6-1). Therefore, we did not consider the combination of Sema3Ai and rehabilitation alone to be effective in the chronic phase of SCI. Additionally, for simplicity, we have focused on whether or not to add additional treatment (Sema3Ai) to the combination of transplantation and rehabilitation, which had been shown to be effective for chronic SCI in rodents in a previous report (Tashiro et al. 2016). In response, we have added a graph depicting the transition in motor function between the combination of rehabilitation and Sema3Ai and rehabilitation alone in the chronic phase of SCI (Figure 6-1) and explanation accordingly.

" The absence of a difference in motor function between rehabilitation with inhibitors and rehabilitation alone during the chronic phase, as suggested by our new data, could also provide support (Figure 6-1)" (line 388-390)

" Extended Figure 6-1 shows a comparison of BBB score with Rehabilitation + Sema3Ai and Rehabilitation." (line 599-600)

" Extended Figure 6-1. Comparison of BBB score with Rehabilitation + Sema3Ai (n=26) and Rehabilitation (n=26)." (line 622-623)

------------------------------------------------------------------------------------------

2) Sema3A1 did not increase the volume of the injury core (Fig 2). This is surprising, as in prior studies (Kaneko 2006) Sema3Ai greatly increase the volume of the epicenter. The authors should discuss this discrepancy, which is probably due to the chronicity of the injury.

(Response)

We thank for the reviewer's comment. We agree with the reviewer that the chronicity of the injury diminished the treatment effect of Sema3Ai to the lesion volume. The other reason we believe that the volume of the injured epicenter did not change with or without Sema3Ai in this study is likely due to the significant improvement in volume of the injured epicenter resulting from transplantation. This comparison was made between individuals which underwent only rehabilitation without transplantation in the new experiment and those that underwent the combined rehabilitation and transplantation in this study (Figure 2-1). This led us to consider that the increase in volume caused by Sema3Ai alone might have been overshadowed. In response, we have added a picture which compared the epicenter tissue of rehabilitation without transplantation in the new experiment and those that underwent the combined rehabilitation and transplantation in this study (Figure 2-1) and explanation accordingly.

" The observation that the volume of the transplanted area did not differ between the presence and absence of the Sema3Ai, contrary to the previous reports (Kaneko et al. 2006), could be influenced by the chronicity of the injured spinal cord which limits the simple treatment effect of Sema3Ai and the substantial increase in volume caused by the transplantation itself as observed in our new data from single rehabilitation in the chronic phase (Figure 2-1)." (line 358-363)

" Extended Figure 2-1 shows an axial-slice of the SCI epicenter of single rehabilitation group." (line 555)

" Extended Figure 2-1. Axial-slice of the SCI epicenter with H&E staining of TR group and single rehabilitation group. Scale bar, 100 μm" (line 614-615)

------------------------------------------------------------------------------------------

3) Figure 2 - what timepoint where the images acquired (2C-D)? Was it at the end of rehab? For readability, this information should be included in the figure legend and the Results text.

(Response)

We thank for the reviewer's comment and apologize for insufficient explanation. All images except Fig. 2A were acquired at DPI 110 when all the rehabilitation training have finished. In response, we have added explanation accordingly.

" To evaluate the effect of transplantation on the SCI epicenter after all rehabilitation (DPI 110), hematoxylin and eosin (H&E) staining was used to confirm the gross appearance (Fig. 2B)." (line 214-215)

" C, Comparison of the SCI epicenter volume with H&E staining at 110 DPI TSR (n=4) and TR (n=4), p=0.72. D, Axial-slice IHC of the SCI epicenter with HNA and Ki67 at 110 DPI." (line 556-557)

------------------------------------------------------------------------------------------

4) Regarding statistics, ANOVA should be used with a post-hoc test (Fig 6), not a t-test, to account for the multiple comparisons.

(Response)

We thank for the reviewer's comment. In response, we have changed a post-hoc test with multiple comparisons and changed text accordingly.

" A, BBB score comparison. TSR (n=15) and TR (n=15). Repeated-measures ANOVA assuming sphericity. p=0.018. Tukey multiple comparisons of means: 53 DPI, p=0.013; 60 DPI, p=0.14, 67 DPI, p=0.15, 74 DPI, p=0.20, 81 DPI, p=0.14, 88 DPI, p=0.016; 95 DPI, p=0.0072; 102 DPI, p=0.07; 109 DPI, p=0.0031." (line 597-599)

------------------------------------------------------------------------------------------

5) There should be more explanation for why the graft sends such few axons, and this should compared with prior literature.

(Response)

We thank for the reviewer's comment. In this study, axonal elongation of host origin was conspicuous, while that of transplanted cells was not. There have been no previous studies examining the origin of axonal elongation in experiments where NS/PCs were transplanted after 42 days post-injury, as suggested as a sufficient duration for the chronic phase by Kwon et al., (2013) with rehabilitation. In the paper on SCI with chronic phase rehabilitation, Tashiro et al., (2015) did not evaluate, and Shibata et al., (2023) also did not examine how much the axons derived from transplanted cells increased. As for papers involving transplants, Karimi-Abdolrezaee et al. (2010), Kumamaru et al. (2013), Kawabata et al. (2016) did not evaluate graft derived axons either. In the paper on SCI in chronic phase without rehabilitation, Hashimoto et al. (2023) have shown the existence of graft-derived axons through immunohistochemistry using STEM121. In this perspective, this research has confirmed STEM121 at the SCI epicenter in the Fig. 4D, H either. However, it's important to note that STEM121 is not specific to axons. Lu et al. (2012) evaluated graft-derived axons by labeling transplanted NSCs with GFP in subacute phase. However, this was not done in this study because it would have affected other evaluations. Although this is outside the scope of the current study, further investigation is needed to determine what is important for the axonal elongation of graft-derived axons and what kind of changes would be induced if the present system, using a Semaphorin3A inhibitor, is applied to it. In response, we have added limitation accordingly.

" Limitation

In this study, we observed host-specific axon outgrowth, which was attributed to the presence of specific antibodies targeting these axons. Notably, previous research has not investigated the origin of axon elongation in scenarios where neural stem/progenitor cells (NS/PCs) were transplanted 42 days after injury. This time frame, recommended by Kwon et al. (2013), is considered sufficient to enter the chronic phase of rehabilitation. In their study on spinal cord injury (SCI) during the chronic phase of rehabilitation, Tashiro et al. (2015) did not evaluate the extent of axon growth from transplanted cells. Similarly, Shibata et al. (2023) also overlooked this aspect in their investigation. As for the papers with transplants, Karimi-Abdolrezaee et al. (2010), Kumamaru et al. (2013) and, Kawabata et al. (2016) also did not evaluate transplant derived axons either. In the paper on SCI in chronic phase without rehabilitation, Hashimoto et al., (2023) have shown the existence of graft-derived axons through immunohistochemistry using STEM121. In this regard, the present study has also confirmed STEM121 at the SCI epicenter in the Figure 4D, H and Figure 4-1. However, it's important to note that STEM121 is not specific for axons. Although the transplanted cells were NSCs in the subacute phase, Lu et al., (2012) evaluated graft-derived axons by labeling the transplanted cells with GFP. However, this was not done in this study because it would have interfered with other evaluations. Further investigation is needed to determine what is important for the axonal extension of graft-derived axons and what kind of changes would be induced when the present system, using a Semaphorin3A inhibitor, is applied to it in the future studies." (line 395-413)

" Karimi-Abdolrezaee, S., et al. (2010). "Synergistic effects of transplanted adult neural stem/progenitor cells, chondroitinase, and growth factors promote functional repair and plasticity of the chronically injured spinal cord." Journal of Neuroscience 30(5): 1657-1676." (line 452-454)

" Kawabata, S., et al. (2016). "Grafted Human iPS Cell-Derived Oligodendrocyte Precursor Cells Contribute to Robust Remyelination of Demyelinated Axons after Spinal Cord Injury." Stem Cell Reports 6(1): 1-8." (line 457-459)

" Kumamaru, H., et al. (2013). "Therapeutic Activities of Engrafted Neural Stem/Precursor Cells Are Not Dormant in the Chronically Injured Spinal Cord." Stem Cells 31(8): 1535-1547." (line 465-466)

" Lu, P., et al. (2012). "Long-Distance Growth and Connectivity of Neural Stem Cells after Severe Spinal Cord Injury." Cell 150(6): 1264-1273." (line 469-470)

" Extended Figure 4-1 shows graft axon expansion with STEM121 at the SCI epicenter." (line 587)

" Extended Figure 4-1. Graft axon expansion with STEM121 at the SCI epicenter. A, Graft axon expansion with STEM121 in TSR group at the SCI epicenter.in sagittal direction. Scale bar, 100 μm. B, Graft axon expansion comparison with STEM121 volume TSR (n=4) and TR (n=4), p=0.62. The comparison was tested with a two-sample t-test. *p<0.05, **p<0.01, ***p<0.005." (line 617-620)

------------------------------------------------------------------------------------------

6) The lack of effect on MEP amplitude is surprising, given the increased number of axons crossing the site of injury, justified as phase cancellation due to different conduction velocities. However, this is difficult to believe due to lack of differences in the latency.

(Response)

Thank you for your insightful comment. We would like to address the MEP changes as follows. Regarding latency, although the injury in this study was severe, the original nerve fibers were not completely damaged. Therefore, the presence of a few remaining original fibers, which exhibit similar latencies in both groups, should not raise concerns. Additionally, any treatment might have modified the function of these fibers. Conversely, a greater number of damaged fibers, which were sensitive to the treatments, restored conduction action potential and elongated durations post-intervention. However, the latencies were, in any case, less than those seen in infarctions.

------------------------------------------------------------------------------------------

Minor:

P1 L1: 'bur' should be 'but'

P4 L58: 'Sema3A' should be 'Sema3Ai'

P11 L245: 5-HT staining needs to be introduced better

Figures should include dorsal-ventral, rostral-caudal, left-write arrows to improve readability.

(Response)

We thank for the reviewer's comment. Accordingly, we have corrected the text as suggested, and arrows were incorporated into the figures.
