# Supplementary figures and images for: Chronic Spinal Cord Injury Regeneration with Combined Therapy Comprising Neural Stem/Progenitor Cell Transplantation, Rehabilitation, and Semaphorin 3A Inhibitor
Source: eNeuro. 2024 Feb 9;11(2):ENEURO.0378-23.2024. doi: 10.1523/ENEURO.0378-23.2024 (PMC10866332; doi:10.1523/ENEURO.0378-23.2024)

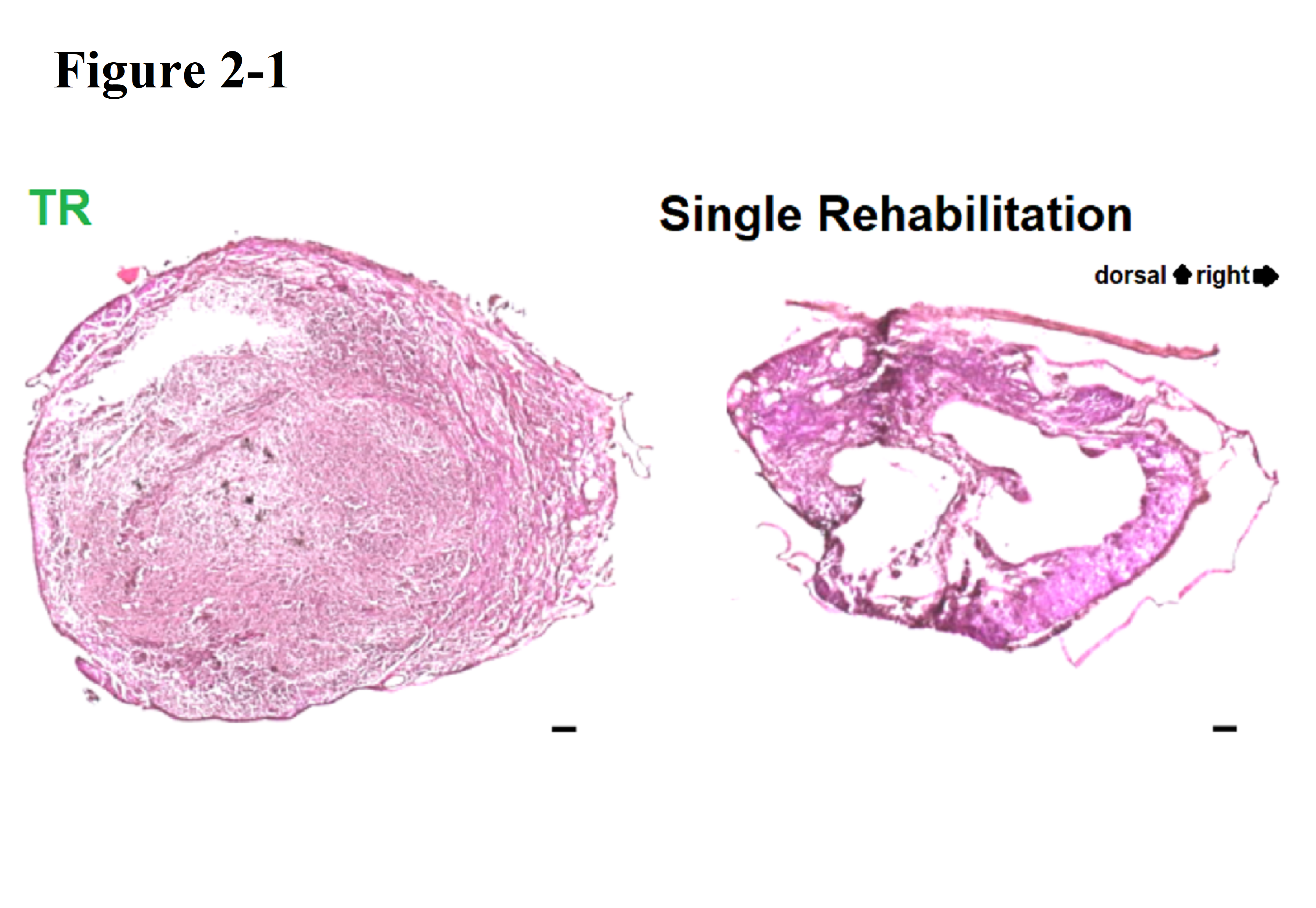

Supplement: Figure 2-1 — Axial-slice of the SCI epicenter with H&E staining of TR group and single rehabilitation group. Scale bar, 100 μm. Download Figure 2-1, TIF file. [file eneuro-11-ENEURO.0378-23.2024-s001.tif]

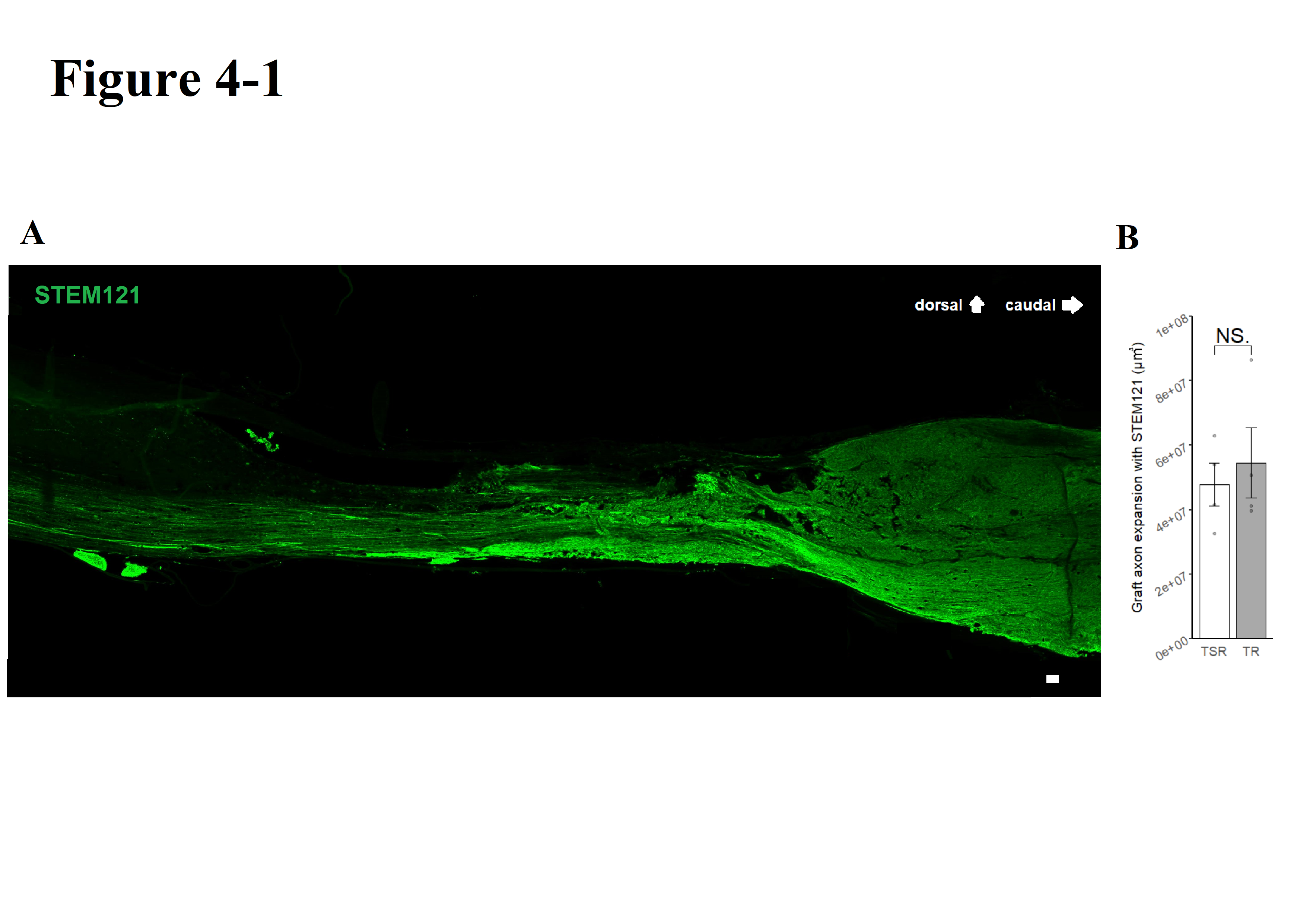

Supplement: Figure 4-1 — Graft axon expansion with STEM121 at the SCI epicenter. A, Graft axon expansion with STEM121 in TSR group at the SCI epicenter.in sagittal direction. Scale bar, 100 μm B, Graft axon expansion comparison with STEM121 volume TSR (n=4) and TR (n=4), p=0.62. The comparison was tested with a two-sample t-test. *p<0.05, **p<0.01, ***p<0.005. Download Figure 4-1, TIF file. [file eneuro-11-ENEURO.0378-23.2024-s002.tif]

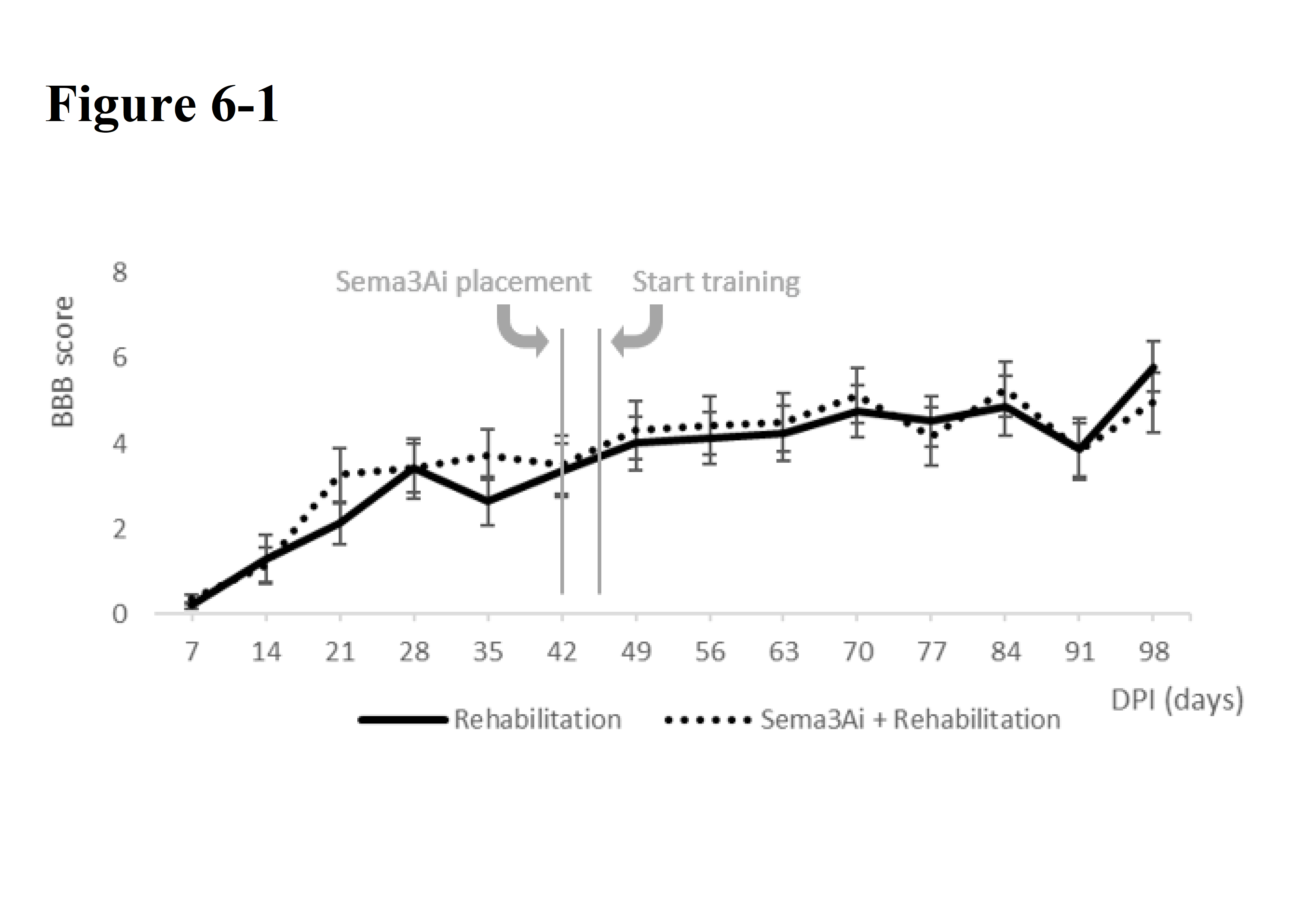

Supplement: Figure 6-1 — Comparison of BBB score with Rehabilitation + Sema3Ai (n=26) and Rehabilitation (n=26). Download Figure 6-1, TIF file. [file eneuro-11-ENEURO.0378-23.2024-s003.tif]
